# Computed tomography-guided vertebroplasty using a stereotactic guidance system (stereo-guide)

**DOI:** 10.4103/2152-7806.63904

**Published:** 2010-05-31

**Authors:** Nancy E. Epstein

**Affiliations:** Clinical Professor of Neurosurgery, The Albert Einstein College of Medicine, Bronx, NY, USA and Chief of Neurosurgical Spine and Education, Winthrop University Hospital, Mineola, NY 11501, USA

## THE BENEFITS OF THE “STERO-GUIDE”

This technical note entitled “Computed tomography (CT)-guided vertebroplasty using a stereotactic guidance system [stereo-guide]” focuses on the utilization of this protractor with CT guidance in the radiology suite to perform percutaneous vertebroplasty with greater safety and accuracy. Their protractor consisted of a flat plastic block with “deeply grooved protractor markings at 5° intervals” (range from 0° to 30°). While in the CT scanner, the device was placed on the patient's back, and the needle was optimally directed through the center of the pedicle through the appropriate groove. It was successfully employed in nine patients undergoing 10 procedures involving either the thoracic or lumbar spine; there were no complications. Furthermore, the CT scanner additionally facilitated monitoring the injection of vertebroplasty material into the vertebral body; cement consolidation and the presence/absence of extravasation could be immediately assessed. The authors should be commended for devising this protractor that appears to be a useful adjunct when performing percutaneous vertebroplasty in the radiology suite utilizing CT guidance.

## LIMITATIONS OF INTRAOPERATIVE FLUOROSCOPY

Previously, when vertebroplasties were performed under fluoroscopic guidance either in the radiology suite by neuroradiologists/radiologists or in the operating room by spine surgeons, localizing the correct level, particularly in the thoracic spine, was often difficult. Another limitation was the inability to “accurately” document extravasation of material into the spinal canal, disc space, etc. (as compared with the use of the CT scan in the radiology suite). This, therefore, increased the risk of inadvertently injecting more material, causing further neural and/or other injuires.

## PROS FOR THE ISO-C IN THE OPERATING ROOM

Although other studies have routinely employed CT-guidance in radiology suites to perform vertebroplasty/kyphoplasty (VK), one unique report focused on the safety and efficacy of utilizing the Isocentric C-arm fluoroscopic cone beam CT (Iso-C) in the operating room.[6] In this report, the Iso-C provided not only high spatial resolution, but also real-time 3D-reconstruted images for performing five different spinal procedures including a vertebroplasty, a transoral cervical vertebral biopsy, a thoracic vertebral body biopsy, drainage of a pelvic paraspinal/epidural abscess, and placement of a paraspinal marker for tumor localization. 

## FREQUENCY OF OSTEOPOROTIC COMPRESSION FRACTURES

One of the major concerns regarding the performance of vertebroplasty is the advanced age of the patients involved, and their increased number of attendant comorbidities making them poor candidates for “open” operations. Osteoporotic compression fractures of vertebral bodies occur in 5-20% of patients between the ages of 50 and 80, and are four times more frequent in females.[2] Their frequency is estimated at 1.6 million compared with 420,000 for strokes, 365,000 for myocardial infarctions, and 250,00 for Breast Cancer.

## COMPLICATIONS OF VERTEBROPLASTY/KYPHOPLASTY

### Neurological deficits attributed to cement leakage

Vertebroplasty and kyphoplasty can also cause severe neurological injury in an estimated of 1-19% of cases utilizing polymethylmethacrylate (PMMA) or Cortoss (OrthoVita, Malvern, PA, USA).[3–5] In Patel's series of 14 patients undergoing VK, averaging 74.9 years of age, 6 developed new neurological deficits within less than 24 h, while 8 deteriorated within 37 postprocedure days [3-112 days].[5]Notably, 12 patients required “open” operations to address these deficits. In one series using Cortoss, the risk of adverse events (primarily extravasation) remained high (19.1%), while other complications included re-fracturing (13%), and adjacent level fractures (43.4%).[3] Another series utilizing Cortoss, cited a 38% rate of "asymptomatic leakage," with four patients (11.8%) showing significant complications including pulmonary embolism, radiculopathy, rash, and cord compression.[4]

Nevertheless, with malignant disease, the risks of neurological injury from VK are mitigated by the patients' limited long-term prognoses. In a series involving 74 vertebrae in 51 patients with terminal metastatic disease or multiple myeloma, 15 (29%) had incomplete/complete cord compression before vertebroplasty and sustained no further deficits; only one patient developed a new cauda equina syndrome within 48 h.[8] Otherwise, analgesia was successful in 94% of patients on day 1, 86% at 1 month, and 92% (in 11 of 12 surviving patients) at 1 year.[8]

### Adjacent-level fractures following VK

An added concern is the increased risk for adjacent-level fractures following VK. In one study, 2 of 25 patients treated with VK compared with none managed conservatively developed delayed adjacent-level fractures.[7] In another series, 20 of 106 patients (18.9%) developed adjacent-level factures within 24 months of VK procedures.[1]

### Careful patient selection and long-term outcomes of VK procedures

Given the risks/complications associated with VK procedures utilizing any modality, we should reexamine patient selection based upon short- and long-term outcomes. In a randomized prospective study, 50 patients with acute/subacute osteoporotic compression fractures (<8 weeks old) were treated either with percutaneous vertebroplasty or conservative management.[7] Within 1 month, the vertebroplasty group exhibited a “significantly lower” Visual Analog Scale [VAS] when compared to those managed conservatively, but there was no significant difference in VAS-documented pain at 3 and 12 months.
